# Data on mineral composition, fatty acids, oxidative stability, UV-VIS spectra and fluorescence emission of the Dersani® and Sunflower® oils used as a cicatrizing agent

**DOI:** 10.1016/j.dib.2019.104427

**Published:** 2019-08-22

**Authors:** Joseane Bortolanza de Oliveira, Flavio Santana Michels, Elaine Silva de Pádua Melo, Carlos Eduardo Domingues Nazário, Anderson Rodrigues Lima Caires, Daniel Araujo Gonçalves, Claudia Andrea Lima Cardoso, Valter Aragão do Nascimento

**Affiliations:** a Group of Spectroscopy and Bioinformatics Applied Biodiversity and Health (GEBABS), School of Medicine, Federal University of Mato Grosso do Sul, Campo Grande/MS, Brazil, S/N, Campo Grande, 79070-900, Brazil; b Optics and Photonics Group, Institute of Physics, Federal University of Mato Grosso do Sul, Campo Grande, Brazil; c Federal University of Mato Grosso do Sul, Institute of Chemistry, Campo Grande, Brazil; d Department of Chemistry, Minas Gerais State University – UEMG, Ituiutaba, Brazil; e Centro de Estudos Em Recursos Naturais, UEMS, Dourados, MS, Brazil

**Keywords:** Essential, Sunflower oil, Stability, Antioxidants, Spectrophotometry

## Abstract

Dersani® and sunflower® oils are used by the Brazilian population as a cicatrizing agent. However, data on physical and chemical properties of these oils are scarce. In this data article on oils, we determined a total of 14 fatty acids composition by gas chromatography (GC), as well as quantifying the elements contents (Ca, K, Mg, Al, Cr, Fe, Mn, Na, P, Se and Zn) using inductively coupled plasma optical spectrometry (ICP OES). Rancimat method was used to determine the oxidative stability of the oils at temperature of 110 °C, in which the induction times for Dersani® and Sunflower® oils were 1.54 (±0.02) and 6.21 (±0.17) hours, respectively. Spectroscopic techniques UV-VIS and fluorescence were employed to obtain spectral datasets. UV-VIS and fluorescence spectroscopy reveals the presence of phenolic, tocopherols, tocotrienols and methyl-linolenate compounds in the oils. The determination of mineral and others contents in oils is an important criterion for the assessment of oil quality with regard to oxidation and their toxicity, properties and storage.

**Table:** Specifications Table

Subject area	Biochemistry
More specific subject area	Essential fatty acid oil and thermal stability
Type of data	Table, Figures
How data was acquired	Chromatograph model 3800 (Varian, Walnut Creek, USA); Microwave digestion System (Speedwave four, Berghof, Eningen, BW, Germany); ICP OES (iCAP 6300 Duo, Thermo Fisher Scientific, Bremen, Germany); Rancimat 893 (Metrohm Co, Basel); Spectrophotometer (Operating Range: 190 - 1100 nm, Lambda265UV/Vis, Perkin Elmer, Waltham, MA, USA); Spectrofluorometer (FS-2, Scinco, Seoul, Korea);
Data format	Raw, Analyzed
Experimental factors	a) An amount of the oil (0.16 g for Dersani® and 0.16 g for Sunflower, respectively), was added in a solution of KOH (5%) in MeOH (4 mL) and heated in a water-bath at 100 °C for 5 min. A solution of NH_4_Cl–H_2_SO_4_ in MeOH (5.0 mL) was then added and homogenized for 30 seconds. b) Sample Pretreatment with Microwave-Assisted Techniques: Weigh 0.7 g of the each sample into the digestion vessel. Add 6.0 mL of HNO_3_ (65%, Merck - Darmstadt, Germany) and 2.0 mL of H_2_O_2_ (30%, Merck - Darmstadt, Germany). c) Preparation of the oil samples to analysis by Rancimat: Samples of 3.0 ± 0.1 g of oils with volatile oxidation products were stripped from the oil and dissolved in deionized water. d) Analysis by UV/VIS: Samples of the Dersani® and Sunflower® oils were diluted in HPLC grade hexane at a concentration of 10 g/L. e) Analysis by Fluorescence: The samples were diluted in HPLC grade hexane at a concentration of 10 g/L.
Experimental features	The gas chromatography method allowed the determination of 14 fatty acids. Determination of concentration levels of contents (Al, Ca, Cr, Fe, K, Mg, Mn, Na, P, Se and Zn) in Dersani® and sunflower® oils used in the treatment of wounds. Induction times were determined on each sample in a Metrohm Rancimat. Identification of phenolic compounds and tocopherols in Dersani® and Sunflower® oils using UV-VIS spectroscopy. Identification of molecular groups in Dersani® (Tocopherols, tocotrienols) and Sunflower® (alpha-tocopherol and methyl-linolenate) oils using fluorescence spectroscopy.
Data source location	City of Campo Grande, State of Mato Grosso do Sul, Brazil.
Data accessibility	The raw data was attached to this data article as Supplementary Material.

**Table:** 

**Value of the data** • Data on the oxidative stability of essential fatty acid oil can be compared with the international standard EN14112 for samples of sunflower and vegetable oil, as well as others oils used in the treatment of wounds. • Data on the presence or absence of the level of metals such as Cu, Fe, Mn and Ni are involved in the oxidation of oils. Studies should be performed considering other types of oils used in the treatment of wounds. • The datasets obtained from different techniques, lead to different results respectively for Dersani® oil and Sunflower® oil. The study would a lead to new questions and investigations of oils used in the treatment of wounds. • This data are important for monitoring purposes and also to fill the gaps in two oils used to treatment of wounds.

## 1 Data

Dersani® oil is used to treat wounds and is composed of essential fatty acids [1]. On the other hand, cold-pressed sunflower seed oil (*Helianthus annus*) has been used topically to improve the skin barrier and prevent systemic infections [2].

**Fig. 1:**
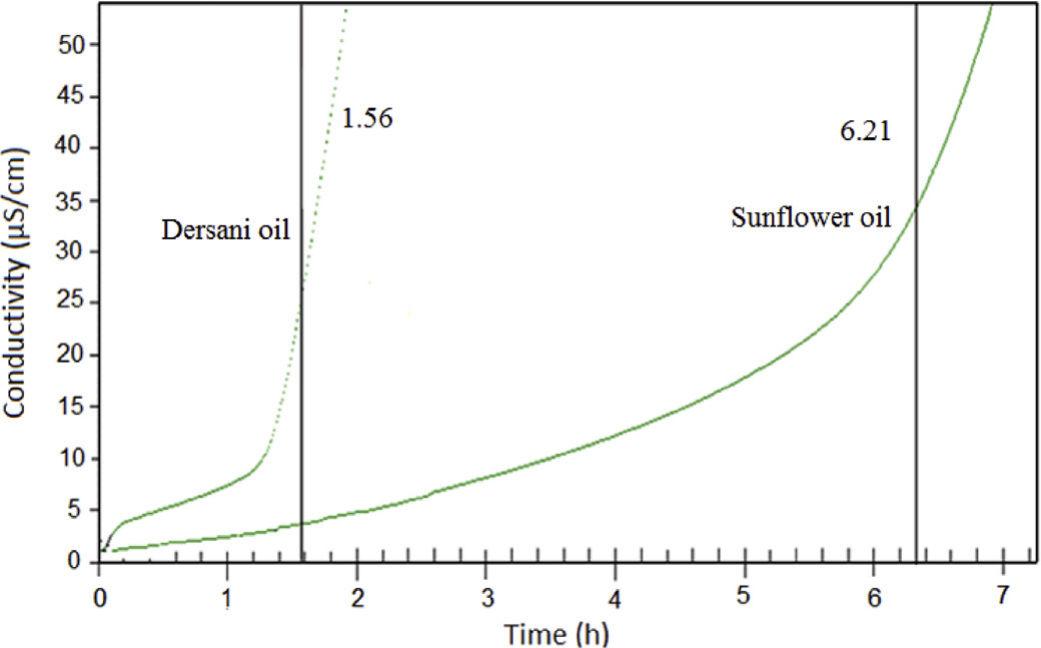
Conductivity versus time determined by the Rancimat method. Oxidation stability of oils at 110 °C. The induction time of Dersani® and Sunflower® oils were 1.56 and 6.21 hours.

**Fig. 2:**
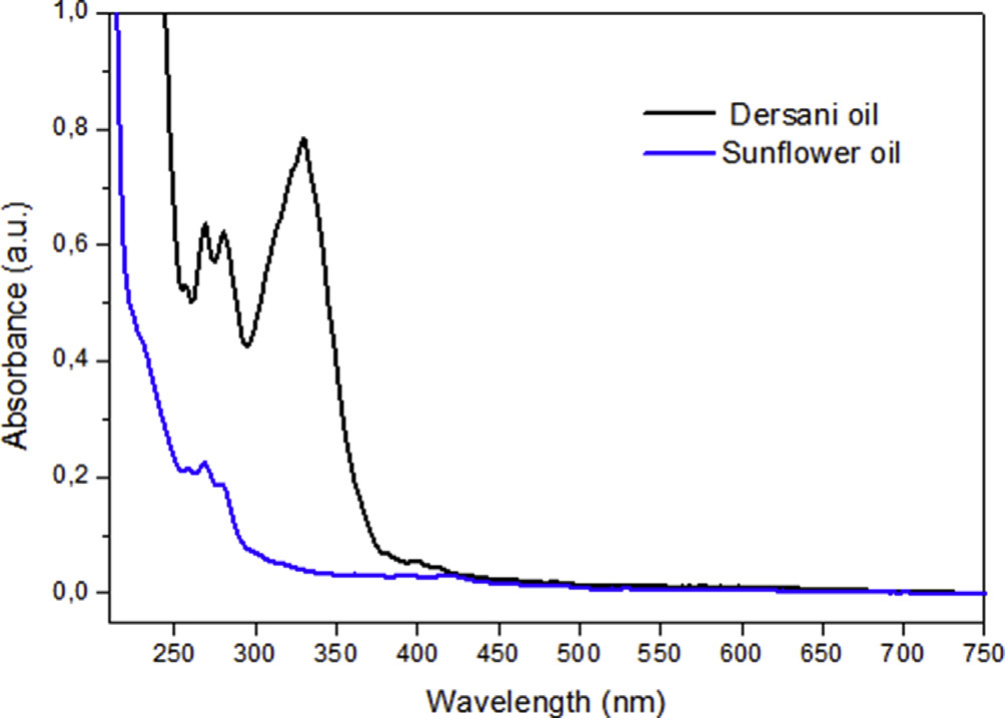
UV-vis absorption spectra of absorbance versus wavelength from 200–750 nm for the Dersani® and Sunflower® oils.

**Fig. 3:**
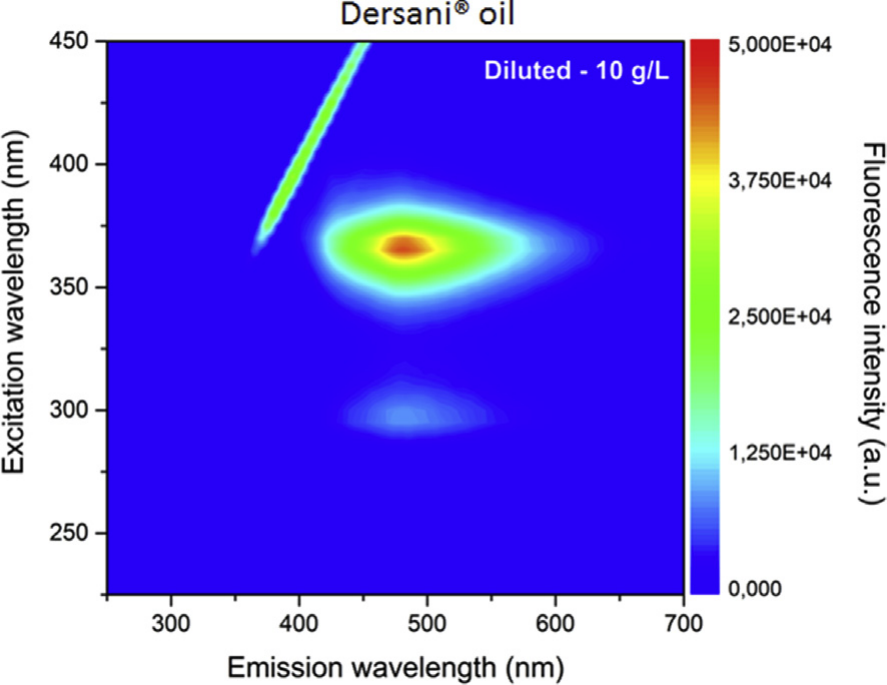
Excitation–emission map of Dersani® oil obtained by exciting between 225 and 450 nm and emission in the 250–700 nm range.

**Fig. 4:**
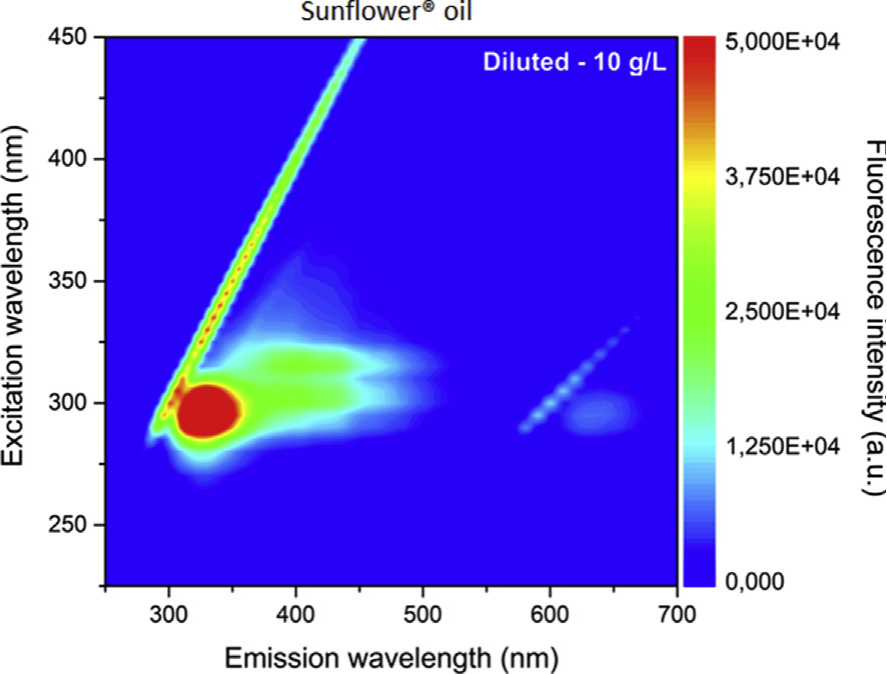
Excitation–emission map of Sunflower® oil obtained by exciting between 225 and 450 nm and emission in the 250–700 nm range.

The manuscript is organized as follow: the data presented in subsection 1.1 (Table 1) include results on fatty acids composition of oils by CG analysis. In subsection 1.2 (Table 2) we presented data on elemental content in Dersani® and Sunflower® oils detected by ICP OES. The subsection 1.3 (Table 3, Fig. 1) provides data on oxidative stability obtained by Rancimat method. In the subsection 1.4 (Fig. 2), data on UV-VIS are presented as a graph of absorbance versus wavelength. The subsection 1.5 (Fig. 3, Fig. 4) includes the data on excitation and emission map for fluorescence of the Dersani® and sunflower® oils.

**Table 1:** Fatty acids composition of Dersani® and Sunflower® oils.

Fatty acids (%)	Dersani®	Sunflower®
Capryc (C10:0)	0.12	0.12
Lauric (C12:0)	0.11	0.11
Myristic (C14:0)	0.18	0.17
Palmitic (C16:0)	6.89	7.02
Palmitoleic (C16:1)	0.32	0.3
Stearic (C18:0)	2.67	2.74
Oleic (C18:1)	32.18	32.44
Linoleic (C18:2)	54.81	54.41
Linolenic (C18:3)	0.32	0.31
Arachidic (C20:0)	0.24	0.26
Gondoic (C20:1)	0.46	0.45
Behenic (C22:0)	0.98	0.95
Erucic (C22:1)	0.44	0.45
Lignoceric (C24:0)	0.28	0.28
SFA	11.47	11.65
MUFA	33.4	33.65
PUFA	54.81	54.72

**Table 2:** Analytical data on elemental content present in the Dersani® and Sunflower® oils detected in ICP OES (in units of mg/Kg ± standard deviation of triplicate).

Elements	Dersani® Oil (mg/Kg)	Sunflower® Oil (mg/Kg)
Al	10.30 ± 0.03	118.20 ± 0.40
Ca	41.00 ± 0.60	28.25 ± 0.04
Co	< LOQ	< LOQ
Cr	3.60 ± 0.11	3.45 ± 0.07
Cu	< LOQ	< LOQ
Fe	1.30 ± 0.02	1.40 ± 0.04
K	11.00 ± 0.12	46.50 ± 0.05
Mg	22.50 ± 0.14	15.04 ± 0.03
Mn	1.30 ± 0.02	< LOQ
Na	243.50 ± 27.80	58.20 ± 0.10
Ni	<LOQ	<LOQ
P	127.50 ± 4.40	169.50 ± 1.23
S	< LOQ	< LOQ
Se	0.90 ± 0.08	2.00 ± 0.01
Zn	2.35 ± 0.08	1.60 ± 0.01

<LOQ - Analyte concentrations were below the limits of detection.

**Table 3:** Rancimat at 110 °C of the Dersani® and Sunflower® oils.

	Dersani® oil	Sunflower® oil
Racimat [h]	1.56 ± 0.02	6.21 ± 0.17

Mean ± SD: Standard deviation values are expressed as mean of samples analyzes in triplicates.

### 1.1 Data obtained by analysis by CG

Data on chromatographic analysis used to estimate the percentages of saturated fatty acids (SAT), monounsaturated fatty acids (MUFA) and polyunsaturated fatty acids (PUFA) of the oils examined in this article are in Table 1.

### 1.2 Data analysis by ICP OES

In Table 2, a total of 11 elements (Al, Ca, Cr, Fe, K, Mg, Mn, Na, P, Se and Zn) were quantified in the Dersani® and Sunflower® oils. The data in Table 2 show that Co, Cu, Ni and S level was below limit of detection in the samples. The experiments were analyzed by ICP OES after digestion procedures (Table 4, Table 5).

**Table 4:** Operating program for the microwave digestion system.

Step	Power (W)	Temperature (°C)	Hold Time (min)	Ramp time (min)	Pressure (Bar)
1	1305	150	10	5	35
2	1305	250	15	3	35
3	0	50	10	1	35
4	0	50	0	0	35
5	0	50	0	0	35

**Table 5:** Analytical characteristics of ICP–OES method: Limit of detection (LODs), limit of quantification (LOQs) and correlation coefficient (*R*^*2*^*)*.

Elements	LOD (mg L^−1^)	LOQ (mg L^−1^)	*R*^*2*^
Al	0.003	0.010	0.999
Ca	0.02	0.08	0.999
Co	0.0002	0.0008	0.999
Cr	0.0004	0.001	0.999
Cu	0.002	0.006	0.999
Fe	0.0009	0.003	0.999
K	0.01	0.04	0.999
Mg	0.01	0.03	0.999
Mn	0.0001	0.0004	0.999
Na	0.0001	0.0005	0.999
Ni	0.002	0.005	0.999
P	0.005	0.02	0.999
Se	0.001	0.005	0.999
Zn	0.0006	0.002	0.999

### 1.3 Data on thermal stability obtained from Rancimat

The oxidative stability index of oils (induction period time) was given in Table 3. The Rancimat induction time at 110 °C varied from 1.56 ± 0.02 to Dersani® oil and 6.21 ± 0.17 h to Girassol® oil (Table 3). The Fig. 1 shows the example of an analysis the induction time of 1.56 h to Dersani oil and 6.21 h to sunflower oil.

### 1.4 Data on UV-VIS

Fig. 2 shows typical plots of UV-Vis absorption spectra of the two oils from 200 to 750 nm. All oils have two absorption peaks from 250 to 350 nm. Dersani® oil shows a band centered near 330 nm and low intensity bands between 260 and 300 nm. Sunflower oil showed bands already known at approximately 232 and 270 nm, which are used for monitoring the degradation products in vegetable oils. In this selected wavelength range the absorption of phenolic compounds (270–330 nm) and tocopherols (325 nm) occurs, although the contribution of some fatty acids should not be excluded.

### 1.5 Data on fluorescence

The Fig. 3, Fig. 4 shows the excitation-emission map of Dersani® and Sunflower® oils obtained by exciting at 225–450 nm and collecting the emission in the range 250–700 nm. The oils show differences in their fluorescence spectra.

The emission of Dersani® oil occurs in the region near 500 nm for excitation wavelengths at 370 nm. Such emission can be associated with the presentation of vitamin E, a group of natural antioxidants composed of tocopherols and tocotrienols. On the other hand, the emission bands of Sunflower® oil may be derived from the presence of alpha-tocopherol which when excited near 300 nm should emit at 325 nm, and methyl-linolenate with emission around 400 nm when excited at 300 and 320 nm.

## 2 Experimental design, materials and methods

### 2.1 Material

Samples of the two most popular brands of essential oils were purchased at local pharmacies in the city of Campo Grande, Brazil. Total ten samples of one brand of each sample were taken for study. In this way total 20 samples (10 samples for Dersani [Manufacturer: Laboratório Daudt Oliveira Ltda] and 10 sunflower oil [Manufacturer: Phytotratha] respectively) were collected for study considering the same batch reference number and date of manufacture.

### 2.2 Chromatographic method

The sample preparation procedure was performed according to Ref. [3] as follow: a) an amount of 0.16 g of Dersani oil and 0.16 g of sunflower oil was weighed separately, and for the esterification process 4 ml of KOH (5%) in MeOH was added to each sample; b) samples were placed in a thermal bath with boiling water for 5 minutes; c) after cooling, 5 mL of NH_4_Cl–H_2_SO_4_–MeOH (0.5:10:89.5 w:v:v) was added in each oil sample, which were homogenized on a vortex mixer (Labnet International S0200 VX-200 Vortex Mixer with Combi head) for 30 seconds and placed in boiling water for 5 minutes; d) after cooling, each sample was individually homogenized after the addition of 4 ml of saturated NaCl solution; e) subsequently, 5 ml of hexane was added to the oil samples and then homogenized again using a vortex mixer; f) a quantity of 1 μL of each sample was used by the chromatography equipment.

The analysis of fatty acid composition by gas chromatography was performed on a Thermo Fisher Scientific (FOCUS GC) chromatograph equipped with a flame ionization detector (FID) and manual injector, capillary column DB-Wax (30 m length, 0.32 mm internal diameter, and 0.25 μm of film, J & W Scientific). The injector temperature was programmed at 250 °C; column temperature at 180 °C for 20 min, and ramp rate of 2 °C/min up to 220 °C; temperature of the detector at 260 °C. Hydrogen gas was used as the mobile phase at a rate of 1.0 mL/min. “Make up nitrogen” gas flow at a flow of 20 mL/min was used to minimize band broadening and injection volume of 1 μL. For the identification of fatty acids, the retention times were compared with those of the methyl ester standards (Sigma-Aldrich), while the quantification was performed by area normalization, expressing the result in percentage of area of each acid over the total area of fatty acids (%).

### 2.3 Microwave-assisted digestion

A microwave system (Speedwave four®, Berghof, Germany) was used for digestion of samples. About 0.7 g of each samples were weighted, transferred inside Teflon vessels and 6 ml of HNO_3_ (65%, Merck - Darmstadt, Germany) and 2 ml of H_2_O_2_ (30%, Merck - Darmstadt, Germany) were added. All the samples were digested in triplicates. The microwave instrumental parameters and conditions used for oils are reported in Table 4, respectively. A blank solutions were prepared using 6 ml deionized water (18 MΩ cm) obtained from a Milli-Q Plus system (Millipore, Bedford, MA, USA), with 6 ml of concentrated HNO_3_ (65%, Merck - Darmstadt, Germany) and 2 ml of concentrated H_2_O_2_ (30%, Merck - Darmstadt, Germany).

### 2.4 Process of data analysis by ICP OES

Analysis were carried out using an inductively coupled plasma optical emission spectrometry model iCAP 6300 (Thermo Fisher Scientific, Bremen, Germany) with axial configuration. The operating conditions of the ICP-OES equipment were 1250 W of a RF power, 12 L.min^−1^ of a plasma flow rate, 0.5 L.min^−1^ of an auxiliary gas flow rate, 0.45 L.min^−1^ of a nebulizer flow rate and 20 s of stabilization time. The analytical emission lines (nm) were determined by the operational software iTEVA of ICP OES instrument as follows: Al (396.100 nm); Ca (422.673 nm); Co (228.616 nm); Cr (267.716 nm); Cu (324.754 nm); Fe (259.940 nm); K (766.490 nm); Mg (279.553 nm); Mn (257.610 nm); Na (588.995 nm); Ni (221.647 nm); P (214.914 nm); Cu (327.396 nm); Se (196.09 nm) and Zn (213.856 nm). High purity argon 99,996% (White-Martins-Praxair, MS, Brazil) was used to purge the optics and to form the plasma.

A multi-element stock solutions (Specsol, São Paulo, Brazil) containing 100 mg.L^−1^ of each elements (Al, Ca, Co, Cr, Cu, Fe, K, Mg, Mn, Na, Ni, P, Se and Zn) was used to prepare the calibration solution by sequential solution with ultrapure water (18 MΩcm). To determine the concentration of elements in both oils, calibration curves were built on seven different concentrations. All determinations were done in triplicate. A blank was carried out in the same way. From the resources of the ITEVA software that controls ICP OES, we checked the small region of the spectrum to determine if a line for a given element is free of interference from lines of other elements.

The detection limit (LODs) and quantification limit (LOQs) was calculated according to IUPAC [4]. They are calculated as follows: LOD = 3*stdError/A1; LOQ = 10*stdError/A1, where stdError = the standard error of estimation of the background signal and A1 = gain coefficient of the calibration curve. *R*^*2*^ is the square of the correlation coefficient. A perfect line would have an *R*^*2*^ value of 1, and most *R*^*2*^ values for calibration curves are over 0.999 for all analytes (see Table 5).

### 2.5 Process of data analysis by Rancimat

The oxidative stability of the oils was evaluated by mean of the Rancimat method. Stability was expressed as the oxidative induction period (IP, hrs) measured at 110 °C on a Rancimat 893 (Metrohm Co, Basel) and air flow of 10 L/hr. Samples of 3.0 ± 0.1 g of oil with a Volatile oxidation products were stripped from the oil and dissolved in deionized water. The increased of conductivity was expressed through a curve from which the induction period can be calculated by the interception of tangent to the inclination and the tangent to the curve level part. Thus, the time taken to reach a level of conductivity was measured (see Fig. 1). The methodology adopted was performed according to the European standard EN14112 and according to the guidance of the National Agency of Petroleum [5], Natural Gas and Biofuels (ANP) [6]. For both oils, analyzes were performed in triplicate.

### 2.6 Process of data analysis by UV/VIS

Samples of the Dersani® and Sunflower® oils were diluted separately in HPLC grade hexane at a concentration of 10 g/L. UV-visible absorption measurements were performed using a spectrophotometer (Lambda 265UV/Vis, PerkinElmer, Waltham, MA, USA) and an optical cuvette made from quartz with a 10 mm optical path and with four polished faces was used as a sample holder. The UV-visible absorption spectra were collected in the 200–750 nm range.

### 2.7 Process of data analysis by fluorescence

Excitation-emission matrix fluorescence spectra were measured using a spectrofluorometer (FS-2, Scinco, Seoul, Korea). The excitation–emission matrices of fluorescence were obtained by exciting the samples in the wavelengths from 225 to 450 nm in 5 nm steps and collecting the emission between 250 and 700 nm in 1 nm steps. The excitation and emission slits were 5 nm and the sensitivity of the detector was 600 V. The samples were diluted in HPLC grade hexane at a concentration of 10 g/L. A four-sided quartz cell with 10 mm optical path was used. A four-sided quartz cell with 10 mm optical path was used.

## References

[1] Ferreira A.M., de Souza B.M.V., Rigotti M.A., Loureiro M.R.D. (2012). The use of fatty acids in wound care: an integrative review of the Brazilian literature. Rev. Esc. Enferm. USP.

[2] Stoia M., Oancea S. (2015). Selected evidence-based health benefits of topically applied sunflower oil. App. Sci. Rep..

[3] Maia E.L., Rodriguez-Amaya D.B. (1993). Avaliação de um método simples e econômico para a metilação de ácidos graxos com lipídios de diversas espécies de peixes. Rev. do Inst. Adolfo Lutz São Paulo.

[4] Long G.L., Winefordner J.D. (1983). Limit of detection: a closer look at the IUPAC definition. Anal. Chem..

[5] European Committee for Standardization (2003). EN 14112:2003 - fat and oil derivatives - fatty acid methyl esters (FAME), Determination of oxidation stability (accelerated oxidation test). https://www.techstreet.com/mss/products/preview/1928580.

[6] Agência Nacional de Petróleo (ANP), Gás Natural e Bicombustíveis, available at: http://www.anp.gov.br/petro/biodiesel.asp (accessed in 30 August 2019).

